# FACTORS ASSOCIATED WITH LONGITUDINAL DIGITAL SURVEY ENGAGEMENT IN THE ELECTRONIC FRAMINGHAM HEART STUDY

**DOI:** 10.1093/geroni/igae098.3974

**Published:** 2024-12-31

**Authors:** Yuankai Zhang, Chathurangi Pathiravasan, Jian Rong, Belinda Borrelli, Jamie Faro, Xuzhi Wang, Chunyu Liu, Joanne Murabito

**Affiliations:** Boston University School of Public Health, Boston, Massachusetts, United States; Boston University School of Public Health, Boston, Massachusetts, United States; Boston University Chobanian & Avedisian School of Medicine, Boston, Massachusetts, United States; Boston University Henry M. Goldman School of Dental Medicine, Boston, Massachusetts, United States; University of Massachusetts Chan Medical School, Worcester, Massachusetts, United States; Boston University School of Public Health, Boston, Massachusetts, United States; Boston University School of Public Health, Boston, Massachusetts, United States; Boston University Chobanian & Avedisian School of Medicine, Boston, Massachusetts, United States

## Abstract

Smartphone surveys are increasingly used in health research, but factors affecting long-term survey response among older adults, are not well understood. We conducted a study of 611 participants (mean age 74 years, 57% women, 86% White) from the electronic Framingham Heart Study to identify sociodemographic, health-related, and technological factors associated with survey response over 32 weeks assessed longitudinally across four 8-week periods. The same number of surveys were sent to the participants with one group receiving half of surveys every 2 weeks and the other group receiving all surveys every 4 weeks. The outcome was the proportion of surveys each participant returned during each period (up to 22 surveys of 20 unique types were sent during each period). Multivariable mixed-effects regressions with random intercepts examined associations between multiple factors and response rates, adjusting for age group (≤75 versus >75 years), sex, and time periods. Lower depressive symptom score (20-item CES-D; β=-0.04, p-value=0.028), younger age group (≤75 years; β=0.08, p-value=0.009), and use of iPhone (vs. Android; β=0.24, p-value< 0.001) were significantly associated with higher response rates. Significant effect modification by time periods (p-value< 0.05) indicated that over time, non-drinkers, iPhone users, and the 2-week survey group had less decline in response rates compared to their counterparts. Findings suggest that sociodemographic, health-related, and technological factors significantly influence digital survey engagement among older adults, with interactions between these factors and time. This highlights the need for targeted strategies to improve long-term engagement, particularly among older participants and those with higher depressive symptom scores.

